# Rapid Review of Interventions Designed to Enhance Personalised Care for People With Dementia When There Are Concerns About Reduced Awareness of Difficulties

**DOI:** 10.1002/gps.70153

**Published:** 2025-09-08

**Authors:** Catherine M. Alexander, Hannah Earle, Anthony Martyr, Linda Clare

**Affiliations:** ^1^ REACH: The Centre for Research in Ageing and Cognitive Health University of Exeter Medical School Exeter UK; ^2^ NIHR Applied Research Collaboration South West Peninsula Exeter UK; ^3^ NIHR Policy Research Unit in Dementia and Neurodegeneration Exeter (DeNPRU Exeter) Exeter UK

**Keywords:** alzheimer, anosognosia, care‐giver, denial, insight, person‐centred, strategy, support, training

## Abstract

**Objectives:**

Awareness of difficulties varies in people with dementia. Low awareness, also termed anosognosia, has been implicated in carer stress and safety concerns, and can be a barrier to effective clinical communication. Little is known about how to manage situations arising from low awareness. This review looked for evidence of existing interventions to enhance care in situations regarding low awareness, and considered their utility, feasibility and acceptability when delivering personalised care.

**Methods:**

We used systematic review methodology, adapted for a rapid timeline, searching five databases and grey literature sources. The review built on an earlier scoping review about measuring awareness in dementia. The protocol was registered on PROSPERO (CRD42024626367). Interventions were included if targeted at people with any dementia type in any setting, or dyads or informal carers, or clinicians. Interventions of any type were eligible where awareness had been measured and addressed, and quantitative outcome data were available. Risk of bias of included articles was assessed. The review is reported as a narrative synthesis.

**Results:**

From the database search, 6042 articles were screened, with additional findings from grey literature. Seven articles were included, describing heterogenous interventions. Two interventions aimed to enhance awareness as the primary goal. No intervention was aimed at informal carers or clinicians, and none addressed specific everyday concerns arising from low awareness. Five non‐pharmacological interventions used methods involving music, a garden, a cognitive programme, interview‐based psychosocial approaches or staff training. These appeared generally acceptable to care recipients, with some feasibility of use, but with limited efficacy. Intervention goals regarding awareness were poorly defined. Outcomes on awareness were mixed in comparison with control groups, with slowing of decline at best. Some improvement in mood, quality of life and coping was observed. Two drug interventions showed a reduction in neuropsychiatric symptoms but limited utility regarding awareness. Available public guidance about awareness issues is relevant but lacks a clear evidence‐base.

**Conclusions:**

The review identified evidence gaps for suitable interventions for managing low awareness in dementia. Existing interventions have limited efficacy and application regarding awareness. There is scope for further intervention development in this area.

## Key Points

- There is a lack of interventions to enhance personalised care in dementia when there are challenges arising from reduced awareness of difficulties.
- Existing interventions lack suitability for managing or negotiating everyday problems around awareness.
- There are no suitable interventions for informal carers or clinicians.
- Guidance provided for managing awareness issues is relevant for family members and carers of people with dementia, but without a clear evidence base.

## 1 Introduction

### 1.1 Rationale

Awareness varies in people with dementia, with some people appearing more aware of dementia‐related difficulties than others [1, 2]. Differing degrees of awareness can be apparent across domains of functioning. For example, self‐awareness about thinking ability, also known as metacognition [3], may not extend to awareness of changes in ability to manage everyday tasks [4]. If awareness is understood as having a reasonable or realistic appreciation of one's situation [5], we can describe disturbances of awareness where there are unrealistic perceptions of one's abilities or difficulties [6]. Typically, these may be overestimation of abilities and/or underestimation of difficulties and can be considered as reduced or low awareness. Reduced awareness is sometimes termed ‘anosognosia’ or ‘lack of insight’ [7]. Denial of difficulties as a psychological reaction to a challenging situation can also manifest as lack of awareness [8]. Reduced awareness can have implications for people living with dementia [2, 9] and carers [10, 11], but also for social care providers and clinicians [12, 13]. As highlighted by this research, reduced awareness is of particular concern if it impairs adequate achievement of daily activities, an important concern for people with dementia, or impedes safety, good relationships, effective communication and provision of appropriate support.

Prevalence estimates of awareness problems in dementia vary widely [14, 15], and may depend on the concept or method of assessing awareness being used [16]; there is no agreed gold standard [7]. Low awareness is more commonly associated with more severe dementia, but at any stage, there is a spectrum of awareness if closely observed [2, 6, 17]. Apparent lack of awareness in early stages may improve over time [18], perhaps due to education or information provision, and adjustment to dementia.

Currently in UK dementia care, awareness is not formally considered in clinical assessments, and there is little information about supportive interventions to improve care in situations where low awareness is problematic. Recent reviews about awareness in dementia focus on psychosocial or neural correlates [19, 20, 21], concepts of awareness [22], or methods of assessment [7, 23]. Reviews of interventions to support people with dementia and/or carers rarely, if ever, discuss awareness [24, 25, 26, 27, 28].

In this review, we looked for information about existing interventions regarding low awareness in people with dementia. An earlier scoping review of literature on measuring awareness [7] revealed a small number of relevant studies describing interventions. Using systematic review methodology adapted for a rapid timeline, we built on those findings and searched research literature since that date (4th February 2019). In addition, we searched clinical guidelines, carer support and health service policy documents, without date restriction.

### 1.2 Objectives

In this review we aimed to identify and appraise interventions designed to enhance personalised care for people with dementia, when there is apparent low awareness of difficulties. The following research questions were addressed:

1. Are there any evidence‐based interventions to enhance personalised care for people with dementia, when there is apparent low awareness of difficulties?
2. What is the utility, feasibility and acceptability of existing interventions for delivery in established health and social care networks?

## 2 Methods

The review followed systematic review methodology [29, 30] adapted for a more rapid review within a limited timeframe [31]. The review protocol is available on PROSPERO at https://www.crd.york.ac.uk/prospero/display_record.php?ID=CRD42024626367.

### 2.1 Eligibility Criteria

#### 2.1.1 Population

Included interventions targeted at people with any type of dementia in any setting, or addressing dyads, or informal carers, or professional carers or clinicians involved in the care of people with dementia.

#### 2.1.2 Intervention

Included interventions for mitigating or resolving problems arising from low awareness or lack of insight or denial of difficulties by the person with dementia, including specific components from a multi‐component intervention if separately identifiable from non‐relevant components. Interventions of any type were eligible, including psychosocial and/or pharmacological interventions, and training and educational programs, aimed at people with dementia, informal or professional carers, or clinicians. We excluded interventions where awareness was not measured, or not addressed by the intervention, or where awareness was measured only as a predictor of a different outcome.

#### 2.1.3 Type of Studies

Included all types of intervention studies presenting quantitative outcome data. Policy documents, reports, and guidelines that discuss management of awareness problems were included and investigated for evidence. Secondary searches in grey literature and public documents provided by key organisations were examined for information on best practice and supporting evidence. We excluded solely qualitative research, articles not available in English, and non‐original articles not specified in the inclusion criteria, that is, editorials, commentaries, letters, book chapters, etc.

*Outcomes* were recorded if measured using validated instruments concerning the welfare of people with dementia and carers, and other relevant outcomes as reported by the included studies.

### 2.2 Information Sources

Primary searches were conducted on 22 November 2024. These comprised the databases MEDLINE, EMBASE and PsycINFO using the Ovid platform, and Web of Science Core collection, for research published over the previous 5 years, since February 2019. We added articles published prior to that date that were included in our earlier scoping review [7]. The scoping review searched these databases in 2019 using compatible search terms, and the added articles were rescreened for interventions. In addition, we searched Social Policy and Practice via the Ovid platform with no date limits.

Supplementary searches included reference checking from relevant retrieved reviews, and citation chasing on Scopus or Google Scholar for eligible articles and relevant conference abstracts from the primary search.

Secondary searches for non‐peer‐reviewed literature from ProQuest Dissertations and Theses via Web of Science were conducted on 22 November 2024, with limited searches on agency websites via Google Advanced Search schema completed on 14 January 2025. Websites searched were those for Alzheimer's Association (USA), Alzheimer's Disease International, Alzheimer's Research UK, Alzheimer's Society (UK), Dementia UK, and World Health Organization, selected after pilot searches for relevant documents from websites of 24 representative organisations worldwide.

### 2.3 Search Strategy

The search strategy was developed using the criteria outlined above. Search terms were guided by relevant existing reviews and keywords and synonyms from relevant published studies. The strategy was peer reviewed and revised in consultation with experts in dementia research and/or evidence synthesis. Key search terms were ‘dementia’ AND ‘awareness’ AND ‘intervention’, with multiple free text synonyms used. In addition, subject headings were used when available for ‘dementia’ and ‘intervention’, selecting subheadings for each database which were considered relevant to the objectives of this review. Terms were searched in title, abstract and keyword (or equivalent) fields; see Supporting Information S1: Table 1 for examples of the search strategies used.

### 2.4 Selection Process

Two reviewers independently screened titles and abstracts for 20% of the articles selected randomly, and checked for agreement, using Rayyan [32]. Initial dual screening showed excellent agreement (98%); subsequently single screening was applied. Full text screening was carried out by both reviewers using EndNote. Decisions where there was uncertainty about inclusion were resolved through discussion or through advice from co‐authors. For articles authored by a member of the research team, the author was not involved in inclusion decisions or appraisal of the study.

### 2.5 Data Extraction

After initial piloting of data extraction using an Excel spreadsheet, two reviewers manually extracted data from the included studies, with discussion to resolve any differences. Attempts were made to contact authors of recent studies where feasible, for information not provided in the article.

Data was extracted to describe the included article, that is, authors, publication date and country of study, number of participants, population, type of intervention, underpinning theory (if stated). Characteristics of the interventions were recorded using the Template for Intervention Description and Replication (TIDieR) checklist [33, 34]. Outcomes were recorded focusing on measures regarding awareness and the welfare of the person with dementia and/or the carer.

### 2.6 Risk of Bias Assessment

We used the Evidence Project Risk of Bias Tool [35] to assess each included study. Two reviewers performed the risk of bias assessment, reaching agreement through discussion.

### 2.7 Synthesis

The review is reported in a narrative synthesis. Due to the variation in study design, measures used, stage of dementia in participants, length of follow‐up, and aims of the studies, quantitative synthesis of the results was not suitable. Outcomes have been appraised to consider the effectiveness of interventions and the importance of the outcomes for people with lived experience and to consider the acceptability, utility and feasibility of the interventions.

### 2.8 Confidence in Cumulative Evidence

The TIDieR checklist was used to compare the completeness of reporting of interventions [33, 34] and to comment on quality of the evidence. Two reviewers independently rated the studies and reached agreement through discussion. A percentage score was calculated for each study to facilitate comparison.

### 2.9 Patient and Public Involvement and Engagement (PPIE)

The PPIE group for this project, comprising three carers and one person with dementia, was involved in discussions at the planning stage, and in interim discussions about the included interventions and outcomes reported. Further discussion about the review conclusions will form a basis for wider stakeholder consultation.

### 2.10 Rapid Review Methods Used

In keeping with guidance for conducting rapid reviews [31], a restricted approach was used in a number of areas to accelerate the review as the available timeframe was limited. The review built on findings from a previous scoping review of studies that measured awareness in people with dementia. For new searches, a limited number of databases was used. Additional restricted searches of grey literature were conducted after primary searches were completed, as the primary search yielded only a small number of studies suitable for inclusion. Dual screening of title/abstracts was limited, having reached satisfactory agreement on the initial sample. In a minor change from the protocol, we proceeded with dual screening of full texts with discussion about included studies, as there were relatively few full texts to screen. Similarly, two reviewers conducted data extraction, risk of bias and quality assessment, as the number of included studies was small enough to allow this.

## 3 Results

### 3.1 Study Selection

**FIGURE 1:**
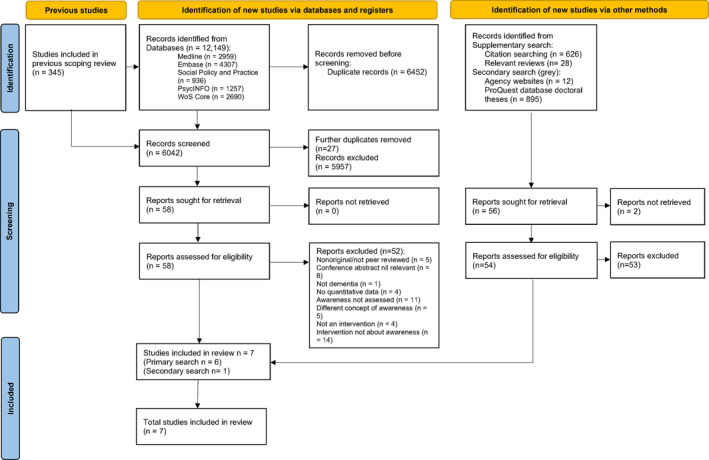
PRISMA flow diagram.

The primary search of five databases found 12,149 articles, from which 6452 duplicates were removed. The addition of 345 articles from the previous scoping review led to screening of 6042 titles and abstracts. There were 58 articles suitable for full text screening, which found six articles meeting inclusion. Supplementary searches of the citations and references from included articles, and relevant reviews, did not lead to additional included articles. Secondary searches of grey literature yielded one relevant PhD thesis, meaning a total of seven documents were included. See PRISMA flow diagram in Figure 1 for details, and Supporting Information S1: Table 2 for information about excluded articles from the primary search.

### 3.2 Study Characteristics

Of the seven included studies, four were community‐based, involving people with mild‐to‐moderate dementia [36, 37, 38, 39]. Two studies involved people with more advanced dementia, in a care home [40] and an in‐patient unit [41]. One study included people with mild or very mild dementia living at home or in long‐term care settings [42]. The studies had a range of designs and were predominantly in preliminary stages of testing the interventions, with no definitive randomised controlled trials found. As such, studies were small with sample sizes ranging from 16 to 65. Six interventions addressed participants with dementia, and one comprised a staff training programme for the benefit of residents with dementia. Most dementia subtypes were Alzheimer's disease, or type not specified; one study included only participants diagnosed with sub‐cortical vascular dementia [38]. Carers were included in outcome assessments for two of the interventions [40, 42], and carer outcomes were reported elsewhere for two studies [37, 38], or reported informally [36]; see Table 1 for more details.

**TABLE 1:** Characteristics of studies.

Author, year and country	Study design and setting	Participants with dementia (*n*)	Dementia stage, subtype (*n*), severity (mean score and SD)	Demographics	Other participants role (*n*)	Intervention description	Control group (and blinding)
				Sex: Female (F); male (M)			
				Age years: Mean (SD)			
Arroyo‐Anllo et al., 2013 [36]. Spain.	Exploratory study with matched groups. Community	*n* = 40 Intervention *n* = 20 Control *n* = 20	Mild to moderate. AD. MMSE: Intervention 19.30 (3.68) Control 19.90 (2.93)	Intervention Sex: 18 F; 2 M Age: 73.48 (3.56) Control Sex: 19 F; 1 M Age: 75.15 (4.23)	Informal carers: Spouse or adult child (presume *n* = 40)	Familiar music stimulation: Listening to self‐selected familiar songs at home, to promote autobiographical memory and enhance self‐awareness.	Listening to a selection of non‐familiar songs. (No information on blinding)
Bertrand et al., 2023 [37]. Brazil.	Pilot RCT Community	*n* = 47 Intervention *n* = 23 Control *n* = 24	Mild to moderate. Dementia not specified. MMSE: Whole sample range 10–24	Intervention Sex: 16 F; 7 M Age: 78.3 (8.4) Control Sex: 13 F; 11 M Age: 77.3 (8.4)	Informal carers: Spouse or adult child (presume *n* = 47).	Cognitive stimulation therapy‐Brasil. Psychosocial group intervention, adapted for Brazilian population. Aim to enhance awareness through implicit learning and multi‐sensory stimulation.	Treatment at usual: Visits to psychogeriatrician every 2–3 months, and ongoing anticholinesterase medication (all participants). (Participant blinding: Not feasible. Researchers: Blind re outcome assessment and data analysis.)
Clare et al., 2013 [40]. Wales, UK.	Pilot RCT with cluster randomisation. Care home	*n* = 65 Intervention *n* = 32 Control *n* = 33	Severe. AD (18), VaD (9), mixed (5), FTD (2) Dementia not specified (31). FAST stage: Intervention stage 6 (*n* = 6) Stage 7 (*n* = 26) control Stage 6 (*n* = 15) Stage 7 (*n* = 18)	Intervention Sex: 32 F; 25 M Age: 82.3 (7.4) Control Sex: 33 F, 26 M Age: 84.6 (8.5)	Care homes (*n* = 8) Staff (*n* = 57): Intervention *n* = 29 Control *n* = 28	AwareCare. Awareness‐based staff training intervention for caring for people with severe dementia in long‐term care settings. Training staff to identify signs of awareness to enable more interaction and stimulation and so enhance well‐being in resident.	Treatment as usual. (Researchers blind re outcome assessments.)
Gueib et al., 2020 [41]. France.	Exploratory observational study In‐patient: Dementia cognitive behavioural unit	*n* = 34 Intervention *n* = 16 Control *n* = 18	Moderate to severe. AD or ADRD. MMSE: Intervention 10.2 (5.5) Control 12.4 (4.8)	Intervention Sex: 11 F; 5 M Age: 82.1 (7.8) Control Sex: 10 F; 8 M Age: 82.4 (6.2)	Not recorded	Art, memory and life healing garden. Direct access from behavioural in‐patient unit to a green space with sensory‐rich environment using nature and art. Aims to enhance cognitive function, well‐being and self‐awareness.	Participants who did not access the garden during the study period. (Participant blinding not feasible. Researcher blinding: No information.)
Hilgeman, 2010 [42]. Alabama, USA.	Feasibility study with block randomisation. Community (*n* = 8) or long term care i.e. assisted living or care home (*n* = 10)	*n* = 18: Recruited as dyads Intervention *n* = 10 Control *n* = 8	Mild or very mild. AD (4), VaD (4), dementia not specified (8), other (2). CDR: Intervention 0.5 (*n* = 4) 1 (*n* = 6) Control 1 (*n* = 8)	Intervention Sex: 7 F; 3 M Age: 80.8 (4.47) Control Sex: 6 F; 2 M Age: 84.25 (7.99)	Informal carers: Spouse (8) Child (8) Niece (1) Paid companion (1)	Preserving identity and planning for advance care intervention (PIPAC). Individual psychosocial intervention combining 2 interview‐based components: Self‐maintaining/Legacy based project, and a self‐adjusting, future planning component (person centred advance care plan) to improve coping and quality of life of people with early dementia.	Emotional support administered by 2 phone calls each lasting 10–20 min, providing empathic listening and reflection. (3 participants seen face to face). (Participant blinding not feasible. Researchers not fully blinded for logistic reasons).
Moretti et al., 2002 [38]. Italy.	Open label pilot study with matched groups. Community	*n* = 16 Intervention *n* = 8 Control *n* = 8	Mild to moderate. Subcortical VaD. MMSE: Intervention 19.68 (3.80) Control 21.83 (3.94)	Intervention Sex: 3 F; 5 M Age: 72.23 (3.46) Control Sex: 3 F; 5 M Age: 72.45 (1.21)	Not recorded	Open‐label drug trial comparing effects of rivastigmine versus low dose aspirin on neuropsychiatric symptoms in people with subcortical vascular dementia	Low dose cardio‐aspirin 100 mg/d. (Participants and physicians non‐blinded.)
Rocca et al., 2007 [39]. Italy.	Retrospective naturalistic study using chart review. Community	*n* = 58 Risperidone *n* = 22 Olanzapine *n* = 16 Quetiapine *n* = 20	Mild to moderate. Probable AD. MMSE: Risperidone 18.92 (3.51) Olanzapine 19.32 (2.88) Quetiapine 19.33 (4.14)	Risperidone Sex: 12 F: 10 M Age: 75.4 (3.96) Olanzapine Sex: 16 F; 0 M Age: 75.6 (3.30) Quetiapine Sex: 8 F; 12 M Age: 74.50 (3.71)	Not recorded	Retrospective drug trial comparing effects of 3 different 2nd generation antipsychotics, risperidone, olanzapine, and quetiapine on behavioural/neuropsychiatric symptoms.	No control group. Comparison groups of 3 different drugs. (Participants and physicians non‐blinded).

Abbreviations: AD, Alzheimer's disease; ADRD, AD related disorder; CDR, clinical dementia rating; F, female; FAST, functional assessment staging; FTD, frontotemporal dementia; M, male; MMSE, mini‐mental state examination. RCT, randomised control trial. VaD, vascular dementia.

### 3.3 Interventions

The intervention designs were varied, and only two aimed to enhance awareness as a primary goal. These two utilised very different methods, of individual familiar music therapy at home [36], and access to an art and nature healing garden during a hospital stay [41]. One study investigated changes in awareness as a secondary outcome in a cognitive stimulation therapy (CST) group trial [37], and another as a secondary outcome for an individual home‐based psychosocial intervention to improve coping and adjustment to dementia [42]. Two studies investigated the impact of medication on neuropsychiatric symptoms (NPS) and behaviour and looked at the impact on awareness as a secondary outcome [38, 39]. One study was centred around the observation of awareness in care home residents with severe dementia, aiming to improve recognition of signs of awareness by care home staff, to optimise care and quality of life (QoL) for residents [40]. See Table 2 for summary of interventions and outcomes.

**TABLE 2:** Summary of interventions and outcomes.

Intervention	Aims	Theory	Measure used and outcome re awareness	Other participant outcomes	Carer outcomes
*Familiar music* Arroyo‐Anllo et al., 2013 [36]	To see if familiar music therapy can enhance self‐awareness, including awareness of condition.	Music therapy known to have benefits on cognition and neuropsychiatric symptoms in dementia. Music memory is often relatively preserved in dementia. This intervention drew on theories that familiar (and self‐selected) music can act as a perceptual cue to trigger autobiographical memories [73], which in turn could enhance self‐awareness.	Measure: SCQ For SCQ total score and the anosognosia subscale: Intervention group maintained awareness over time Control group awareness worsened.	Cognition: No significant impact, although control group worsened over time.	Not measured. Anecdotal reports of benefits of being closer to participant.
*Cognitive Stimulation Therapy‐Brasil* Bertrand et al., 2023 [37]	To see if CST can increase awareness and whether this varies for different domains of awareness.	CST is a widely adopted intervention with some evidence supporting use to improve functioning, cognition and quality of life. This study refers to the cognitive awareness model [74] which suggests that memory training to enhance recollection and consolidation of personal memories could address ‘mnemonic anosognosia’ (lack of knowledge of self‐ability) and so enhance self‐awareness. This provided the rationale for exploring the impact on awareness of CST, which uses reality orientation and multisensory memory stimulation to improve implicit learning through familiarity and intuition [75].	Measure: ASPIDD Total ASPIDD scores showed slight decrease over time (improved awareness) in both groups, medium effect size. No significant group effect. Awareness of cognitive ability slightly better in intervention group, with medium effect of time/group interaction. Other subscales nil significant. NB discrepancy score. Unclear whether changes in score reflect changes in self and/or informant ratings.	QoL no effect self‐rated or informant rated. Depression slightly reduced in intervention group over time, large effect size. Cognition slightly worsened over time, both groups, large effect size. ADL ability slightly improved in intervention group over time, medium effect.	Carer burden: No effect.
*AwareCare staff training* Clare et al., 2013 [40]	To see if training staff to use an observational measure to recognise signs of awareness leads to improved care and increased quality of life for care home residents with severe dementia.	Considers awareness in severe dementia through a biopsychosocial model, in which psychological and social or environmental factors contribute to expression of awareness, alongside neurocognitive factors [5]. In care home settings, awareness at a basic sensory or perceptual level is influenced by cues and stimulation in the environment providing an opportunity to respond and engage in some way, if responses are recognised [76, 77]. Training staff to identify signs of awareness could enable more interaction and stimulation and enhance well‐being in residents. [This intervention was alone in measuring awareness not as an outcome, but as a means to improving other outcomes such as quality of life in the care home residents.]	Measure: AwareCare. Not measured as outcome	QoL improved in intervention group, with medium effect size, when family‐rated, not when staff‐rated. No significant change in well‐being, cognition or behaviour.	Staff well‐being, psychological distress, and attitudes not significantly changed. Quality of care unchanged.
*Art, memory and life healing garden* Gueib et al., 2020 [41]	To see if providing access to a garden designed for people with dementia to interact with art and nature can increase wellbeing and self‐awareness, including awareness of condition.	Developed from theories about the innate attraction of nature [78], how nature can aid recovery from stress [79], and help restore focused attention [80]. Combining nature with art was intended to synergistically increase the experience of interacting with beauty by creating a harmonious space [81]. In theory, this could protect against the depersonalising experience of hospitalisation and enhance self‐awareness.	Measure: SCQ Intervention group small improvements in awareness i.e. small increase in SCQ total score and non‐significant reduction in anosognosia (indicating improved awareness). Control group more impaired on total score and anosognosia subscale at f/up. Statistically significant, unclear whether clinically meaningful.	Neuropsychiatric symptoms reduced for both groups over time. No significant difference between the groups. Depression and cognition no significant changes.	Not measured
*Preserving Identity and Planning for Advance Care* Hilgeman, 2010 [42]	To see if a programme combining a project to produce a legacy output and preparing an advanced care plan can improve coping, well‐being and mood in people with early dementia. 2ry aim was to see how this impacted awareness.	Used a stress process model developed from earlier work on stress and coping [82], and coping in dementia [83], that describes the balance between holding on to the past (self‐maintaining) and adapting to an uncertain future (self‐adjusting). The intervention combines a legacy project which reflects a self‐maintaining component of coping to support self‐identity [84], with advance care planning, representing self‐adjusting to dementia.	Measure: MARS‐MFS Discrepancy score reduced in intervention group but increased in the control group, suggesting improved awareness of memory ability post intervention, with large effect size. Post intervention, participants rated ability slightly lower, and carers rated slightly higher and so closer alignment in ratings. Control group self‐rating and carers ratings showed greater divergence at follow‐up, so discrepancy increased; this appears to be driven by large reduction in carer rating at follow‐up.	Depressive symptoms reduced post‐intervention, with large effect size. Decisional conflict/discomfort about advance care planning lessened, with large effect size. Self‐reported increased use of coping strategies post intervention, with large effect size. QoL mixed findings on different measures. Most convincing is improved family‐rated QoL‐AD post intervention with large effect size.	Carer burden was stable in the intervention group but reduced in the control group: suggesting positive impact of the minimally intrusive supportive phone calls.
*Rivastigmine trial* Moretti et al., 2002 [38]	To see if rivastigmine, an anticholinesterase inhibitor with dual action, can ameliorate symptoms, particularly those due to frontal lobe dysfunction in subcortical vascular dementia, including low awareness of condition.	Hypothesised that this drug might be more effective in dementia subtypes with symptoms and radiological evidence of frontal lobe deterioration, as it has dual inhibitory action on acetylcholinesterase and butyrylcholinesterase with an affinity for frontal regions, in contrast to other anticholinesterase inhibitors. Rivastigmine has previously been effective in Alzheimer's disease but limited success in vascular dementia, despite some evidence of cholinergic impairment in vascular dementia. The trial focused on symptoms mediated through frontal regions, including impaired executive function, personality changes and emotional lability, and lack of awareness. These symptoms are associated with carer stress but with limited options for medical treatment.	Measure: CIRS Marginal changes in awareness of ADL ability which improved slightly in intervention group and reduced slightly in control group. Other domains no significant differences.	Behavioural symptoms significant reduction total score Neuropsychiatric symptoms significant reduction for 3/9 symptoms (anxiety, hallucination, wandering) for intervention group Depression: Neither group changed significantly from baseline, but slight improvement in intervention group and slight worsening in control group created significant difference between the groups. Executive function showed significant improvement in intervention group compared to baseline, and compared to control group, which worsened. ADL ability maintained in intervention group.	Carer stress reduced significantly in intervention group compared to baseline and compared to control group.
*2*_*nd*_ *generation antipsychotics* Rocca et al., 2007 [39]	To compare the efficacy and safety of three second generation antipsychotics, risperidone, olanzapine and quetiapine, when prescribed to treat behavioural/neuropsychiatric symptoms in dementia. Awareness assessed as a 2ry aim.	Behavioural and psychiatric symptoms present significant challenges in dementia care. At the time of this study, there were few long‐term studies on 2nd generation antipsychotics in elderly, few head‐to‐head comparisons, and mixed results on safety. The comparison trial of three second generation antipsychotics prescribed for behavioural symptoms examined efficacy and safety. It is relatively unusual for drug trials to assess awareness; however, awareness impairment has been associated with more severe neuropsychiatric symptoms [85], which may explain the inclusion of awareness assessment in this trial.	Measure: CIRS Awareness of cognitive ability and awareness of disease progression worsened significantly over time, for all groups. No effect on awareness of condition or awareness of ADL ability in any of the groups.	Neuropsychiatric symptoms significantly improved in all groups, and very significantly for symptoms delusions, hallucinations and agitation. ADL ability showed significant improvements across the groups. Cognition no change.	Not measured

Abbreviations: ADL, activities of daily living; ASPIDD, assessment scale of psychosocial impact of the diagnosis of dementia; CIRS, clinical insight rating scale; CST, cognitive stimulation therapy; MARS (MFS), memory awareness rating scale (memory function scale); QoL quality of life; SCQ, self‐consciousness questionnaire.

### 3.4 Theoretical Background to Interventions

The theories behind these diverse interventions range from cognitive theories about awareness in relation to autobiographical memory [36, 37], to those emphasising the psychological and social influences on awareness [40], and coping [42], or the impact of the physical environment on stress management and self‐awareness [41], or used biomedical reasoning [38, 39]; see Table 2.

### 3.5 Risk of Bias in Studies

Regarding patient representativeness, none of the studies used random selection of participants, and three studies did not use random assignment to the intervention [38, 39, 41]. There was incomplete reporting of baseline information to assess equivalence in five studies, although two studies corrected for baseline differences [40, 42]. Overall, none were considered to be at high risk of bias; see Table 3.

**TABLE 3:** Risk of bias of included studies (evidence project risk of bias tool [35]).

	Study design			Participant representativeness			Equivalence of comparison groups	
Study	1. Cohort	2. Control or comparison group	3. Pre/post intervention data	4. Random assignment of participants to intervention	5. Random selection of participants	6. Follow‐up rate ≥ 80%	7. Equivalent sociodemo‐graphics	8. Equivalent at baseline on outcome measures
Arroyo‐Anllo et al., 2013 [36]	Yes	Yes	Yes	Yes	No	No	Yes	Yes
Bertrand et al., 2023 [37]	Yes	Yes	Yes	Yes	No	Yes	Yes	Partial^a^
Clare et al., 2013 [40]	Yes	Yes	Yes	Yes	No	Yes	No^b^	No^b^
Gueib et al., 2020 [41]	Yes	Yes	Yes	No	No	Not recorded	Yes	Yes
Hilgeman, 2010 [42]	Yes	Yes	Yes	Yes	No	Yes	No	No^b^
Moretti, 2002 [38]	Yes	Yes	Yes	No	No	Yes	Yes	Partial^c^
Rocca et al., 2007 [39]	Yes	Yes	Yes	No	No	Not applicable^d^	No^e^	Yes

^a^ ASPIDD baseline values provided in graph form.

^b^ Baseline values controlled in analyses (ANCOVA).

^c^ Baseline values not reported for insight scale items.

^d^ Retrospective study.

^e^ Significant difference on sex and living situation.

### 3.6 Outcomes of Interventions

The intervention outcomes are summarised in Table 2 and Supporting Information S1: Table 3. Additional information for two of the included studies [37, 38] was provided in related papers that did not meet the inclusion criteria [43, 44].

#### 3.6.1 Participant Awareness Outcomes

See Supporting Information S1: Table 4 for further information about the awareness measures used.

##### 3.6.1.1 Interventions That Aimed to Enhance Awareness

These two studies [36, 41] both used the Self‐Consciousness Questionnaire [45] to assess awareness. Neither the familiar music intervention nor the healing garden produced significant improvements in awareness on the total scale or the anosognosia subscale. However, both control groups declined in awareness over time, whilst the intervention groups maintained baseline levels of awareness. The difference in stage of dementia in these two studies is reflected in the differing range of scores recorded, with better awareness scores in the participants with less advanced dementia.

##### 3.6.1.2 Studies Investigating Awareness in Interventions Designed for Another Purpose

The CST trial [37] used the Assessment Scale of Psychosocial Impact of the Diagnosis of Dementia [46] to assess awareness. There was no significant group effect, although total scores decreased over time in both groups, suggesting improved awareness, with a medium interaction effect for the awareness of cognitive ability subscale in the intervention group.

The Preserving Identity and Planning for Advance Care (PIPAC) intervention study [42] used the Memory Awareness Rating Scale‐Memory Function Scale [47] to assess awareness of memory function. The discrepancy decreased over time for the intervention group, suggesting improved awareness of memory function, with a large effect size. The changes in the discrepancy were largely due to changes in the carer ratings; at follow‐up carers in the intervention group gave ratings of improved ability whereas those in the control group rated abilities as worse.

Both drug trials used the Clinical Insight Rating Scale [48] to assess awareness. For the Rivastigmine trial [38], awareness scores in the intervention group improved slightly for all items, and worsened slightly for the control group, however, the group difference was only significant for awareness of cognitive ability. The trial comparing second generation antipsychotics [39] showed awareness of cognitive ability and disease progression significantly worsening over time in all groups.

#### 3.6.2 Other Participant Outcomes

##### 3.6.2.1 Welfare

Five of the included studies assessed the participant's welfare in some way, that is measuring QoL, and/or mood, with more extensive assessments of social/emotional adjustment in the PIPAC study [42]. Results of the impact on QoL were mixed, with improvements seen in only 2 of 3 studies [40, 42] and with family‐rated scales alone. Depressive symptoms improved in 3 of 4 studies [37, 38, 42], emphasised by the worsening of depression in the control group [38]. No significant changes were seen in measures of anxiety [42] or well‐being [40]. One study found more use of coping strategies and less decisional conflict post‐intervention [42].

##### 3.6.2.2 Behaviour and Neuropsychiatric Symptoms

Four studies measured behaviour and/or NPS. The studies involving people with more advanced dementia found a reduction in NPS over time but no group effect [41], or no significant changes in behaviour [40]. However, the studies aiming to address NPS [38, 39] both found significant improvements in behaviour and/or NPS as a result of drug interventions.

##### 3.6.2.3 Cognition

Six studies assessed cognition, with most finding no effect of the intervention apart from one trial [38] which reported significant improvement in executive function in the related paper [44]. In another study, cognition significantly deteriorated over time in the control group in contrast to the intervention group where cognition remained stable [36].

##### 3.6.2.4 Activities of Daily Living (ADL)

Three studies looked at ADL, with a non‐significant improvement after CST [37], maintenance of baseline functioning with rivastigmine [44], and a significant improvement in basic and instrumental ADL after treatment with second generation antipsychotics [39].

#### 3.6.3 Carer Outcomes

Carer outcomes were assessed in four of the interventions. There was no significant change in carer burden following CST or PIPAC interventions, although a reduction in burden was seen in the PIPAC control group [42]. Carer stress was significantly reduced in the Rivastigmine trial [38]. No significant changes arose following the AwareCare staff training programme, on measures of staff well‐being, distress or attitudes, or on quality of care in the home [40]. Anecdotally, informal carers reported feeling closer to the participant following the familiar music intervention [36].

### 3.7 Quality of Intervention Reporting

Few studies recorded whether the intervention was tailored or modified, items which may not have been applicable to all studies. Nonetheless, the reporting was almost fully complete for the other checklist items across the studies; see Supporting Information S1: Table 5.

### 3.8 Utility, Feasibility and Acceptability of the Interventions

See Table 4 for a summary, which will be discussed further below.

**TABLE 4:** Intervention utility, feasibility, acceptability.

Intervention	Aims	Materials; staff; training; duration and location of intervention	Fidelity issues	Adverse effects	Reported acceptability	Comments
*Familiar music* Arroyo‐Anllo et al., 2013 [36]	To see if familiar music therapy can enhance self‐awareness, including awareness of condition.	*Materials*: Music database with selection of popular songs. Device for playing digital recording. Professional headphones *Staff:* Informal carer supervised individual participant. *Training:* 1 training session for carer. *Duration and location:* 2–4 min per session, at home. 3 sessions/week for 12 weeks.	Carers reported participant moving/dancing when listening to the music, against protocol. Authors acknowledge the activity may have led to spontaneous singing or listening to music outside the sessions, which was not formally recorded but could enhance effects.	Not recorded	It was felt that length of sessions needed review, as the sessions were very brief. Carers reported that the intervention helped them to be closer to the participant.	Low risk and likely low‐cost intervention. Could be adapted for more flexible use. May have helped delay deterioration in awareness.
*CST‐Brasil* Bertrand et al., 2023 [37]	To see if CST can increase awareness and whether this varies for different domains of awareness.	*Materials*: CST‐brasil manual https://cstbrasil.com.br/web/ *Staff:* 3 trained facilitators for group of 5–8 participants. *Training:* Provided by international CST Centre, London. *Duration and location:* 45 min per session, presume at clinic setting. 14 sessions given over 7 weeks (provided 2 sessions on same day each week).	Fidelity not formally measured. The two weekly sessions were provided on same day of week as more convenient for carers. Sessions adapted to group's abilities/severity of dementia (detail not shown).	Not recorded	Reported in Marinho et al., 2021 [43]. High acceptance, demonstrated by willingness to take part and attendance. Mean attendance 12.8 (SD 1.6) sessions.	Established cognitive intervention. Training and commitment might be an obstacle to uptake. No convincing change in awareness.
*AwareCare staff training* Clare et al., 2013 [40]	To see if training staff to use an observational measure to recognise signs of awareness leads to improved care and increased quality of life for care home residents with severe dementia.	*Materials:* AwareCare observational measure https://medicine.exeter.ac.uk/v8media/facultysites/hls/healthandcommunitysciences/documents/AwareCare_observational_measure.pdf. *Staff:* Staff training (which is part of the intervention) provided by an accredited trainer. *Training*: Little information about requirements for accredited trainers. *Duration and location:* 8‐week intervention in care home. Staff training sessions 2 × 90 min in week 1–2. Scheduled observations 6 × 10 min per week, by each staff member in weeks 3–8. Additional staff group supervisions sessions 1 h/fortnight. Individual 30 min weekly staff support sessions as required.	8 staff members left employment before study ended. Differing amounts engagement between care homes, some with limited number observations due to staff illness, differing managerial support or other changes in the home.	Not recorded	Staff were able to use the observational tool satisfactorily, but some care homes managed this better than others.	Designed for real world application to improve care and quality of life for care home residents. Training commitment; unclear whether would need repeat sessions for new staff. Demonstrated some improvement in QoL for residents but mixed findings.
*Art, memory and life healing garden* Gueib et al., 2020 [41]	To see if providing access to a garden designed for people with dementia to interact with art and nature can increase wellbeing and self‐awareness, including awareness of condition.	*Materials:* The healing garden. This was a 4000 m^2^ secure space, directly accessible and visible from the in‐patient unit. Bespoke design with areas of sun and shade. Access to plants, trees, artwork including sculptures, benches and tables. *Staff:* Garden maintained by green spaces dept. Of the organisation. Activities monitored by healthcare staff. *Training:* Garden design based on published recommendations for healing garden and adapted for people with dementia. *Duration and location:* Garden adjacent to in‐patient unit. Free access to the garden during the inpatient stay without time constraints. In this study, minimum of 12 cumulative hours over a 2‐week period.	Not recorded	Not recorded	Not randomised so unclear whether benefits were specific to the self‐selected group who chose to access the garden. Authors acknowledge that cannot say which elements of the garden were beneficial, weather, activity, being away from the ward etc.	Considerable resource required to create and maintain the garden. Low risk intervention with attractive face validity. May have helped delay deterioration in awareness.
*PIPAC* Hilgeman, 2010 [42]	To see if a programme combining a project to produce a legacy output and preparing an advanced care plan can improve coping, well‐being and mood in people with early dementia. Secondary aim was to see how this impacted awareness.	*Materials:* Manualised interview‐based psychosocial intervention. The protocol and the participant notebook are shown in the PhD thesis. Participants required to collect materials for the legacy project, and in this study participants were given $20 each towards materials. *Staff:* 5 interventionists with background in clinical psychology or social work. *Training*: Trained and supervised by clinical psychologist. Training included independent reading, didactic instruction, role play, adverse event monitoring. *Duration and location:* 4 sessions over 4–6 weeks, at home. Each session lasted 30–120 min, average 74.3 min.	Treatment fidelity checklist used. 92.2% delivered as intended. 1 session ended early due to unexpected visitors. Advance care planning component not carried out for 1 participant due to negative reaction. Some participants already had advance care plan in place which may have altered reactions.	One participant with dementia became upset by the advance care planning component and one refused to initiate the legacy component. Both were happy with intervention overall.	Participants appeared happy with the intervention on the whole, if undertaken with some flexibility. Introducing the advance care planning component clearly required sensitivity. Carer involvement was optional. The minimal support intervention may have been less burdensome and hence more acceptable to the carers.	Unclear how important family contact was for the intervention. Training required but could be suitable for practitioners with prior training in post‐diagnostic support. Structured protocol available Could potentially be used as two separate short interventions depending on needs. No clear impact on awareness but may assist adjustment to diagnosis.
*Rivastigmine trial* Moretti et al., 2002 [38]	To see if rivastigmine, an anticholinesterase inhibitor with dual action, can ameliorate symptoms, particularly those due to frontal lobe dysfunction in subcortical vascular dementia, including low awareness of condition.	*Materials:* Rivastigmine initial dose 3 mg/d, increased to 6 mg/d after 4w. Control was cardio‐aspirin 100 mg/d. *Staff:* Usual physician for prescribing and assessment. Carers supervised medication administration at home. *Training:* Not described. Carers monitored adherence and side‐effects. *Duration and location:* Individual medication taken at home. Dose increased after 4 weeks. Follow up assessments at 1, 3, 8, 12, 16 and 22 months.	Not recorded	No serious adverse effects reported. Intervention group: Nausea 62.5% (*n* = 5), muscle contractions 25% (*n* = 2), anorexia 12.5% (*n* = 1), postural hypotension 12.5% (*n* = 1). Syncope 12.5% (*n* = 1); thought to be related to existing medication olanzapine Also reported side‐effects in the control group. Nausea (*n* = 3), anorexia and heartburn (*n* = 2), constipation (*n* = 2), dizziness (*n* = 1).	Reported side effects; nil serious.	This study targeted symptoms in a specific dementia subtype, that in theory might be ameliorated by this approach, and used appropriate outcome measures. Awareness is infrequently assessed in dementia drug trials. See below for current dose and indications below^a^ (British National Formulary April 2025). Currently no Food and Drug Administration approval for cholinesterase inhibitors in vascular dementia [86]. Relatively well tolerated but no consistent evidence that improves global functioning.
*2* ^*nd*^ *generation antipsychotics* Rocca et al., 2007 [39]	To compare the efficacy and safety of three second generation antipsychotics, risperidone, olanzapine and quetiapine, when prescribed to treat behavioural/neuropsychiatric symptoms in dementia. Awareness assessed as a secondary aim.	*Materials:* 6‐month course of 2nd gen antipsychotic. Mean doses (SD): Risperidone 0.82 mg (0.45), olanzapine 5.62 mg (2.14), quetiapine 77.60 mg (35.3). Doses described as within recommended ranges. No information provided on clinical decision making re choice of drug. *Staff:* Usual clinician. *Training:* Medical training. *Duration and location*: Oral medication administered at home, daily. Assessment described at initiation and at 6 months. No detail of interim procedures.	Not applicable (retrospective study)	All 3 drugs showed adverse effects, no significant difference between groups. Common side‐effects included weight gain (31%) also dizziness and sleepiness. 9/58 (15.5%) report falls or injury. Other side‐effects included abnormal gait, constipation, extra‐pyramidal symptoms, and UTI. Retrospective study of patients who completed 6 months treatment, so does not include any who dropped out.	No control group, only comparisons with other antipsychotics. This study reports 15.5% falls or injury.	See current dose and indications below^b^ (British National Formulary April 2025 [87]). Risks are well documented for initiating antipsychotics and long‐term use. Used, with caution, in clinical situations where neuro‐psychiatric symptoms are not helped by non‐pharmacological interventions. Awareness worsened in all treatment groups so not indicated to improve awareness.

^a^ Current dose and indications (British National Formulary April 2025): Rivastigmine indicated for mild‐to‐moderate dementia in Alzheimer's disease or Parkinson's disease dementia. Initially 1.5 mg twice daily, increased to usual dose 3–6 mg twice daily (max dose 6 mg twice daily). Not listed for vascular dementia, although can be used in mixed dementia, whereas low dose aspirin may be prescribed.

^b^ Current dose and indications (British National Formulary April 2025): Risperidone dose in moderate to severe Alzheimer's disease for persistent aggression unresponsive to non‐pharmacological interventions.250mcg twice daily increase to 500mcg twice daily. Max 1 mg twice daily. Olanzapine for control of agitation in elderly 2.5–5 mg once daily, max 20 mg/d. Quetiapine for psychosis in elderly, 50 mg once daily, adjusted in steps of 50 mg/d.

## 4 Discussion

In this rapid review we used systematic methodology to search five databases for recent, original peer‐reviewed research, and selected grey literature, building on an earlier scoping review. Seven studies met our inclusion criteria describing interventions related to awareness in people with dementia, in which awareness was assessed before and after the intervention, and with quantitative outcome data. The review revealed a lack of research on evidence‐based interventions to support personalised care when there are issues around low awareness.

### 4.1 Gaps in Evidence

All included interventions were targeted towards care of people with dementia, but none were exclusively for people showing low awareness, or to address specific everyday problems associated with low awareness. Two interventions were primarily designed to enhance awareness, with the assumption that increasing awareness would be beneficial [36, 41]. A training intervention indirectly addressed problems around awareness by training staff to recognise signs of actual awareness, to increase interaction [40]. The remaining four studies looked at the impact on awareness of interventions that were initially designed to address a different issue [37, 38, 39, 42]. Apart from one staff training programme [40], there were no interventions addressing problems faced by carers or clinicians in situations around low awareness.

### 4.2 Utility, Feasibility and Acceptability of the Interventions

First considering the two interventions that were designed to enhance awareness [36, 41], both involved low risk, pleasant activities, which demonstrated some impact on reducing the decline in awareness over time, although follow‐up time was short, less than 2 weeks from completing the interventions. The familiar music intervention was simple and could be adapted for more flexible use at home. The healing garden would require considerable planning and budget to create, but the addition of the novel components could be considered within an existing hospital or community garden. The degree of improvement in awareness demonstrated in both these studies was small, and clinical meaningfulness is uncertain.

The potential advantages to the person with dementia regarding maintaining or increasing awareness of difficulties remain unproven; better awareness has been associated with poorer self‐reported QoL and more depressed mood [2, 19], but can contribute to better outcomes in other rehabilitative interventions [49, 50]. Poorer awareness has been associated with more severe NPS and more advanced dementia [51], presumably due to progressive neuropathology. However, with the garden intervention [41] participants had fewer NPS over time regardless of exposure, so the intervention lacked evidence of effectiveness for NPS, and reduction in NPS did not explain the improvement in awareness in the intervention group.

Two interventions were designed to enhance care either for care‐home residents with advanced dementia [40], or people with early‐stage dementia [42]. Both were designed to support existing care provision. With appropriate training, each of these interventions could feasibly be incorporated into existing training either for care home staff [40], or practitioners providing post‐diagnostic support [42]. Both studies set out to improve the experience of living with dementia rather than to increase awareness, and results demonstrated modest or large improvements in QoL and/or mood. Adjustment to dementia was evidenced by greater use of coping strategies and less discomfort with advance care planning post intervention [42]. However, QoL improvements were limited to family ratings for both studies, with no improvement when assessed with staff‐rated measures [40] or self‐ratings [42], which highlights the complexities of using QoL measures to assess the impact of interventions [52].

The two drug trials were orientated to improve NPS and were effective in this aim. Rivastigmine was reasonably well tolerated in participants with subcortical vascular dementia and may have had minor impact on increasing awareness [38], as well as showing significant improvement in executive function. However, whilst it is used for Alzheimer's disease or mixed dementia, rivastigmine is not currently approved for use in vascular dementia.

The risks of antipsychotics in dementia are well documented [53], and whilst this trial showed a reduction in NPS [39], there was no improvement in awareness, suggesting a disconnect between NPS and awareness. Side‐effects were considerable, notwithstanding that this was a 6‐month retrospective study which excluded participants who had stopped taking medication for any reason.

Lastly, the use of CST is well‐established as a cognitive intervention and the related paper for this trial reported high willingness to take part and good attendance [43]. However, improvement in awareness was minimal [37], and apart from improved mood, changes in other outcomes were slight. Training requirements and commitment to the programme might be an initial obstacle, but materials are available internationally.

### 4.3 Measuring and Addressing Awareness

In the absence of a gold standard for measurement, comparisons between the studies are difficult as different measures of awareness were used. These may have been investigating different concepts of awareness [7]. Awareness can be defined in broad terms which encompass biopsychosocial factors [5] or neurocognitive or neuropathological terms, distinguishing lack of awareness from psychological denial [8]. In practice, it may not be possible to make this distinction easily. Effective, pragmatic interventions would need to take the presenting situation and address the most pressing needs at that time, while remaining vigilant to the possibility of change in awareness if an individual is adjusting to dementia [18].

### 4.4 Guidelines and Support Materials

Guidelines retrieved in the primary search, for example, from the National Institute for Health and Care Excellence [54], rarely discussed awareness and provided no additional evidence about interventions. Some patient‐facing material and public documents available for carers discuss awareness or denial, for example, from Alzheimer's Society [55], but the reference lists did not reveal additional evidence for relevant interventions. Some guidance draws on findings from palliative care research around denial, with compassionate and sensible recommendations, which in turn acknowledge the lack of evidence‐base for the advice [56]. Others rely on the considerable expertise of practitioners [57], in the absence of published research findings.

### 4.5 Qualitative and Other Research Not Included in the Review

The search revealed some intervention studies that used qualitative methods or did not otherwise meet our inclusion criteria but merit discussion. Among these were interventions that used a psychotherapeutic approach to enable a gradual increase in awareness about the diagnosis of dementia through group work [58, 59]. A case study addressed issues around awareness of driving capability [60]. This described individual psychotherapy for a person with dementia to facilitate awareness of changes leading to agreement to stop driving. Another study described a telephone follow‐up intervention to reduce readmission in people with dementia after hospitalisation and included people with low awareness in the target group [61].

Medication management in dementia can be an area of concern [62] and a source of family conflict [63], with scope for intervention. Strategies of varying efficacy were reported by carers to assist in medication management when a person with dementia showed little awareness of task difficulties [64]. Lack of awareness is a factor in scam susceptibility [65], with impaired decision‐making increasing risks of financial mismanagement in people with dementia [66], and could be a focus for intervention. There is a surprising lack of research about how best to support families and people with dementia with these everyday issues that can have profound consequences for safety and independent living. This might be because situations where awareness can be problematic are wide‐ranging, as disclosed in PPIE discussions, and would require bespoke or multiple strategies. Better awareness in people with dementia has sometimes been demonstrated when asked to give a third‐person perspective on a situation [67, 68]. This approach could potentially provide an opportunity to intervene to enhance awareness, for example, using Virtual Reality simulations of everyday activities observed from a third person perspective [69].

Awareness has been noted as an area affecting clinical communication [70], and doctors' responses have been documented [71], but evidence for effective strategies for clinicians is lacking. This may in part reflect poor delineation of awareness‐related problems for the person with dementia and/or carers and clinicians. Awareness is inconsistently recorded in clinical assessments, although recently published recommendations may improve this [72].

### 4.6 Limitations

Our search criteria resulted in intervention studies that assessed awareness but were not all designed to intervene in awareness difficulties. Comparison between studies was limited as different awareness measures were used, and other outcomes were not uniformly reported. We aimed for a comprehensive search of quantitative studies and were surprised not to identify multicomponent interventions that address awareness in part. It is possible that there are interventions in specific clinical conditions in dementia, such as diabetes care or continence management, that discuss awareness but were not retrieved by our search strategy. The addition of qualitative research might have revealed some additional interventions in development. Our searches excluded book chapters, and secondary searches used a limited selection of agency websites. This was necessary to keep the review manageable in the available time. However, the yield of evidence from 24 key websites in the pilot search was low, with little indication that more extensive searching would be productive. Our earlier scoping review had overlapping search terms but did not include grey literature, and it is possible that older relevant conference abstracts were missed.

We used the TIDieR checklist to comment on quality of reported interventions. The checklist was designed for reporting an intervention study, and questions about modification may be inappropriate in a review for scoring studies in which no modification took place. Cotterill suggests that ‘voice’ and ‘stage of implementation’ are important in reporting [33]: our included studies were reported by researchers or research clinicians, and these were small scale pilot and feasibility studies, or exploratory studies.

## 5 Conclusions

There are few existing interventions that directly address awareness difficulties in people with dementia. Some studies investigated whether awareness can be enhanced. Whilst greater awareness might enable more open communication about the diagnosis and facilitate care planning and goal setting in early dementia, it can have negative consequences for people with dementia regarding mood and QoL and would not always be an appropriate goal. There is a lack of evidence for the type of support or intervention that would help people with dementia and carers or clinicians negotiate low awareness and its consequences, leaving scope for future research and intervention development in this area.

## Author Contributions

CMA: developing the concept for the review and preparing the protocol, developing and piloting the search strategy, conducting searches, title and abstract screening, full text screening, data extraction, risk of bias and quality of reporting assessments, interpretation of results, preparing the draft manuscript. HE: piloting the search strategy, conducting searches, title and abstract screening, full text screening, data extraction, risk of bias and quality of reporting assessment, commenting on and approving the manuscript. AM: commenting on the protocol, reviewing the draft manuscript and approving the final version. LC: advising on the concept for the review and reviewing the protocol, reviewing the draft manuscript and approving the final version.

## Ethics Statement

The authors have nothing to report.

## Conflicts of Interest

The authors declare no conflicts of interest.

## Supporting information


Supporting Information S1

## Data Availability

The data used in this research is publicly available through database searches.
