# Supplementary material for: Rapid Review of Interventions Designed to Enhance Personalised Care for People With Dementia When There Are Concerns About Reduced Awareness of Difficulties
Source: Int J Geriatr Psychiatry. 2025 Sep 8;40(9):e70153. doi: 10.1002/gps.70153 (PMC12417933; doi:10.1002/gps.70153)
Supplement: Supplementary file 1 — Supporting Information S1 [file GPS-40-e70153-s001.docx]

**SUPPLEMENTARY MATERIAL**

**Supplementary Table 1.** Examples of the search strategies used.

| **Search stage** | **Database (platform)** | **Search strategy** |
| --- | --- | --- |
| Primary search | Medline (Ovid) | 1. Dementia, Multi-Infarct/ or Dementia, Vascular/ or Dementia/ or Frontotemporal Dementia/ or AIDS Dementia Complex/  2. (dement* or Alzheimer* or Lewy or Fronto*).ab,kw,ti.  3. 1 or 2  4. Psychosocial Intervention/ or Internet-Based Intervention/  5. (interven* or educat* or train* or support or program* or strateg* or advice or counselling or guid* or tool* or resource or package or "problem solving").ab,kw,ti.  6. 4 or 5  7. ("*therapy" or rehabilitat*).ab,kw,ti.  8. ("feasibility stud*" or "pilot stud*" or trial* or evaluat*).ab,kw,ti.  9. 6 or 7 or 8  10. (aware* or unaware* or anosognosia or insight or denial or metacognit*).ab,kw,ti.  11. 3 and 9 and 10  12. limit 11 to yr="2019 -Current" |
| Primary search | PsycINFO (Ovid) | 1. AIDS Dementia Complex/ or Dementia with Lewy Bodies/ or Senile Dementia/ or Dementia/ or Semantic Dementia/ or Presenile Dementia/ or Vascular Dementia/  2. (dement* or Alzheimer* or Lewy or Fronto*).ab,ti,id.  3. 1 or 2  4. Response to Intervention/ or Group Intervention/ or Family Intervention/ or Intervention/  5. (interven* or educat* or train* or support or program* or strateg* or advice or counselling or guid* or tool* or resource or package or "problem solving").ab,ti,id.  6. 4 or 5  7. ("*therapy" or rehabilitat*).ab,ti,id.  8. ("feasibility stud*" or "pilot stud*" or trial* or evaluat*).ab,ti,id.  9. 6 or 7 or 8  10. (aware* or unaware* or anosognosia or insight or denial or metacognit*).ab,ti,id.  11. 3 and 9 and 10  12. limit 11 to yr="2019 -Current" |
| Secondary search: theses | ProQuest Dissertations and Theses (Web of Science) | 1. TS=((“dement*” or “Alzheimer*” or “Lewy” or “Fronto*”))  2. TS=(("interven*" or "educat*" or "train*" or “support” or "program*" or "strateg*" or “advice” or “counselling” or "guid*" or "tool*" or “resource” or “package” or "problem solving"))  3. TS=(("therapy" or "rehabilitat*"))  4. TS=(("feasibility stud*" or "pilot stud*" or "trial*" or "evaluat*"))  5. #2 OR #3 OR #4  6. TS=((“aware*” or “unaware*” or “anosognosia” or “insight” or “denial” or “metacognit*”))  7. #1 AND #5 AND #6  8. Refine: Doctoral  No publication date limit |
| Secondary search: agency websites | Alzheimer’s Society (Google advanced search) | site:alzheimers.org.uk (awareness OR unaware OR anosognosia OR insight OR denial OR metacognition)filetype:pdf |
| Scoping review 2019 | PsycINFO (Ovid) | 1. dement*.ti. or dement*.ab.  2. alzheimer*.ti. or alzheimer*.ab.  3. 1 or 2  4. (("pick's disease" or "picks disease" or "pick disease") not ("niemann-pick" or "niemann pick")).ti. or (("pick's disease" or "picks disease" or "pick disease") not ("niemann-pick" or "niemann pick")).ab.  5. 3 or 4  6. aware*.ti. or aware*.ab.  7. 5 and 6  8. unaware*.ti. or unaware*.ab.  9. 5 and 8  10. anosognosia.ti. or anosognosia.ab.  11. 5 and 10  12. insight.ti. or insight.ab.  13. 5 and 12  14. denial.ti. or denial.ab.  15. 5 and 14  16. metacognit*.ti. or metacognit*.ab.  17. 5 and 16  18. discrepanc*.ti. or discrepanc*.ab.  19. 18 and 5  20. 7 or 9 or 11 or 13 or 15 or 17 or 19 |

**Supplementary Table 2**. Excluded studies in full-text screening primary search.

| **Exclusion reason** | **Number articles excluded** | **Reference** |
| --- | --- | --- |
| Nonoriginal/not peer reviewed | (n=5) | Brown, 2017 ^1^; Clare, 1999 ^2^; Franklin, 2021 ^3^; Jones, 1997 ^4^; Miesen, 2006 ^5^ |
| Not people with dementia (at least 50% of sample) | (n=1) | Buchwitz, 2020 ^6^ |
| No quantitative outcome data | (n=4) | Arber, 1987 ^7^; Gotell, 2003 ^8^; Tabak, 1996 ^9^; Vespa, 2022 ^10^ |
| Different concept of awareness | (n=9) | Barnes, 2020 ^11^ ; Barnes, 2020 ^12^ ; Department of Health, 2008 ^13^; Department of Health, 2009 ^14^; Dombrowski, 2024 ^15^; Francis-Coad, 2023 ^16^; NICE National Institute for Health and Care Excellence, 2019 ^17^; Pin Tan, 2019 ^18^; Xavier, 2022 ^19^ |
| Awareness not assessed | (n=12) | Bahro,1995 ^20^; Berenbaum, 2020 ^21^; Burns, 2005 ^22^; Hertel, 2019 ^23^ ; Lantz, 1997 ^24^; Marco, 2023 ^25^; Morgan, 2004 ^26^; Nicosia, 2023 ^27^; Oksnebjerg, 2019 ^28^; Punitha, 2024 ^29^; Sedigh, 2024 ^30^; Steiner, 2021 ^31^ |
| Not an intervention | (n=5) | Bonney, 2007 ^32^; Cotrell, 2006 ^33^; Niehoff, 2022 ^34^; Ross, 2024 ^35^; Silva, 2017 ^36^ |
| Intervention not about awareness | (n=16) | Au, 2023 ^37^; Berk, 2019 ^38^; Binetti, 2013 ^39^; Clare, 2002 ^40^ ; Clare, 2004 ^41^; Contador, 2016 ^42^; de Farias, 2021 ^43^; Dutzi, 2019 ^44^; Geurten, 2021 ^45^; Kelly, 2017 ^46^; Koltai, 2001 ^47^; Martinez-Moreno, 2016 ^48^; Salmon, 2024 ^49^; Schapira, 2023 ^50^; Villars, 2013 ^51^; Yokoi, 2017 ^52^ |

**Supplementary Table 3.** Details of intervention outcomes.

| **Intervention** | **Aims** | **Outcome re. awareness**  **Scores: mean (SD)** | **Other participant outcomes** | **Carer or staff outcomes** |
| --- | --- | --- | --- | --- |
| *Familiar music*  Arroyo-Anllo, 2013 ^53^ | To see if familiar music therapy can enhance self-awareness, including awareness of condition. | Measure: SCQ. Higher scores indicate better awareness.  **SCQ Total**  Intervention  Pre: 10.11 (2.44)  Post: 11.21 (3.14), p= .056  Control  Pre: 10.17 (2.1)  Post 8.07 (1.8), p=.038  Control group worsened over time.  Significant pre-post difference between groups, p=.016  **Anosognosia subscale (raw score)**  Intervention  Pre: 2.03 (2.31)  Post: 2.14 (1.96), p > 0.05  Control  Pre:1.99 (2.44)  Post: 1.03 (2.63)  Significant pre-post differences between the groups, p= .042 | **Cognition**: n/s change (MMSE or FAB) in the intervention group, although control group worsened slightly. | Not measured. Anecdotal reports of benefits of being closer to participant. |
| *CST-Brasil*  Bertrand, 2023  ^54^ | To see if CST can increase awareness and whether this varies for different domains of awareness. | Measure: ASPIDD. Higher scores indicate more impaired awareness.  **ASPIDD total**:  ANOVA group: F (1, 45) = 0.38, p = .542, ηp^2^ = .01  ANOVA time: F (1,45) = 4.63, p = .037, ηp^2^ = .09  with an increase in awareness of disease (lower ASPDD scores).  ANOVA interaction: F (1, 45) = 0.44, p = .510, ηp^2^ = .01  **ASPIDD subscale:** Awareness of cognitive ability  ANOVA group: F (1, 45) = 0.21, p = .650, ηp^2^ =< 0.01  ANOVA time: F (1, 45) = 2.09, p = .156, ηp^2^ = .04  ANOVA interaction: F (1, 45) = 5.28, p = .026, ηp^2^ = .10  Intervention group had better cognitive awareness (lower scores on this subscale) post intervention but n/s change in scores for control group.  Other subscales, ANOVA showed n/s main effects or interaction. | Reported in related paper Marinho et al., 2021 ^55^.  **Quality of life** (QoL-AD self-rated and informant rated). ANOVA n/s effects time, group, interaction.  **Depression** (CSSD): ANOVA interaction F (1, 45) = 14.99, p < 0.001, ηp2 = 0.25. Intervention reduced depression. N/s main effects.  **Cognition**: (ADAS-Cog). ANOVA time, F (1,45) =8.57, p=.005, ηp2 = 0.16; cognition worsened over time in both groups, large effect size. Group and interaction n/s.  **ADL** (ADCS-ADL).  ANOVA Interaction F (1, 45) = 4.50, p = 0.039, ηp2 = 0.09; trend for improvement in intervention group only. | Reported in Marinho et al., 2021 ^55^.  **Caregiver burden** (ZBI).  ANOVA n/s effect group, time or interaction. |
| *AwareCare staff training*  Clare, 2013 ^56^ | To see if training staff to use an observational measure to recognise signs of awareness leads to improved care and increased quality of life for care home residents with severe dementia. | Measure: AwareCare. Awareness repeatedly observed during the training intervention but not as an outcome. | **Quality of life**  (Family rated QUALID)  ANCOVA F (1,29) = 5.88, p=0.022, Cohen’s d= 0.72; improved QoL post intervention.  (Staff rated QUALID)  ANCOVA F (1,57) = 0.31, p=0.58  **Well-Being** (PRS)  ANCOVA n/s  **Cognition** (GADS)  ANCOVA n/s  **Behaviour** (BASOLL)  ANCOVA n/s. | *Care-home staff*:  **Well-being** (MBI)  **Psychological distress** (GHQ-12)  **Attitudes towards people with dementia** (ADQ)  *Care home:*  **Quality of care** (DCPA)  ANCOVA: n/s effect of intervention for any of these measures. |
| *Art, memory and life healing garden*  Gueib, 2020  ^57^ | To see if providing access to a garden designed for people with dementia to interact with art and nature can increase wellbeing and self-awareness, including awareness of condition. | Measure: SCQ. Higher scores indicate better awareness.  **SCQ Total**  Intervention  Pre: 7.5 (2.9)  Post: 9.3 (3.1), p =.02  Control  Pre: 8.6 (3.4)  Post: 7.5 (2.9), p=.03  **Anosognosia subscale (corrected score)**  Intervention  Pre: 0.4 (0.4)  Post: 0.6 (0.5), p=.12  Control  Pre: 0.7 (0.6)  Post:0.4 (0.4) p=.03 | **Neuropsychiatric symptoms**  (NPI-NH)  Significant reduction in both groups over time (improved symptoms).  Intervention  Pre: 37  Post: 19  Control  Pre: 29.5  Post: 15  Mean improvement  Intervention –15.3  Control –11.8  N/s difference in NPI-NH improvement between the two groups.  **Depression** (GDS)  **Cognition** (MMSE, FAB, MT86-1α)  N/s difference in all other measures over time, either group. | Not measured |
| *PIPAC*  Hilgeman, 2010 ^58^ | To see if a program combining a project to produce a legacy output and preparing an advanced care plan can improve coping, well-being and mood in people with early dementia. Secondary aim was to see how this impacted awareness. | Measure: MARS (MFS). Higher ratings indicate better perceived ability.  Higher discrepancy indicates lower awareness.  **MARS Self-rating**  Intervention  Pre: 35.10 (6.03)  Post: 34.80 (7.47)  Control  Pre: 31.50 (7.86)  Post: 34.50 (7.37)  **MARS Carer rating**  Intervention  Pre: 20.89 (9.58)  Post: 22.47 (9.89)  Control  Pre: 22.00 (13.52)  Post: 14.50 (8.33)  **MARS discrepancy**  Intervention  Pre: 15.11 (6.66)  Post 12.33 (11.33)  Control  Pre: 8.71 (17.80)  Post: 19.79 (11.01)  ANCOVA: group F (1,13) = 2.25, p = 0.16, ηp^2^ = 0.15 (large effect). | **Quality of life**  (BASQID self-report)  ANCOVA F (1,15) = 1.13, p = 0.31, ηp2 = 0.07, increased QoL intervention group.  (Self-rated QoL-AD)  ANCOVA n/s.  (Family-rated QoL-AD)  F (1,14) = 5.41, *p* =. 04, ηp2= 0.28  **Depression** (CSSD)  ANCOVA F (1,15) = 5.51, p = 0.03, ηp2 = 0.27; less depressive symptoms intervention group.  **Decisional conflict** ANCOVA F (1,14) = 3.74, p = 0.07, ηp2 = 0.21, intervention group reported less overall conflict or discomfort with advance care planning.  **Coping strategies**  ANCOVA F (1,16) = 3.35, p = 0.09, ηp2 = 0.17, intervention group reported more coping strategies post treatment.  **Anxiety** (CSDD)  **Meaning** (Meaning in Life Scale)  **Social engagement** (Modified Minimum Data Set)  **Emotional and anticipated support** (modified items from Krause’s scale)  **Health related QoL** (EQ-5D)  No meaningful effects on these measures. | **Caregiver burden** (CBI)  ANCOVA F (1,13) = 1.64, p = 0.223, ηp² = 0.11 (medium effect size).  Higher but stable stress in intervention group. Control group reduced stress at follow-up. |
| *Rivastigmine trial*  Moretti, 2002  ^59^ | To see if Rivastigmine, an anticholinesterase inhibitor with dual action, can ameliorate symptoms, particularly those due to frontal lobe dysfunction in subcortical vascular dementia, including low awareness of condition. | Measure: CIRS (4 awareness subdomains)  NB paper states lower scores indicate worse symptoms. The original CIR scale has higher scores for more impaired awareness.  **Awareness Condition**  Intervention  Mean change: 0.50 (0.76)  Control  Mean change: -0.25 (0.71) n/s.  **Awareness Cognition**  Intervention  Mean change: 0.38 (0.74)  Control  Mean change: -0.13 (0.35) n/s.  **Awareness Disease progression**  Intervention  Mean change: 0.38 (0.74)  Control  Mean change: -0.25 (0.46) n/s.  **Awareness Functional ability**  Intervention  Mean change: 0.25 (0.46)  Control  Mean change: -0.50 (0.53), p= 0.017  i.e. awareness of functional deficit improved slightly in intervention group and reduced slightly in control group. -0.25 _+ 0.71 NS50 +_ 0.76 -0.25 _+ 0.71 N | *Primary outcomes* cognition, ADL reported in related paper Moretti et al., 2002 ^60^  **Executive function** (TPC) mean change  Intervention 2.1 (1.1), p= <.01 change compared to baseline.  Control -0.9 (0.6), p=<.05 change compared to baseline.  Significant improvement over control p=<.001.  *Secondary outcomes:*  **Depression** (CSSD) mean change  (higher scores more depression)  Intervention improved by 1.25.  Control deteriorated by 2.28, p=.02 between groups.  N/s changes either group from baseline.  **Behaviour** (BEHAVE-AD) mean change  Intervention - 6.38  Control 7.13, p=0.001, showing improvement in intervention group.  **Neuropsychiatric symptoms** (NPI) mean change  Anxiety:  Intervention -1.8 (1.49)  Control 3.0 (1.41) p=.001  Hallucinations:  Intervention -0.8 (0.89)  Control 1.0 (1.07), p =.005  Wandering:  Intervention 0.1 (0.35)  Control 1.1 (0.83) p = .014  i.e. significant improvement post intervention for 3 symptoms. N/s for remaining 9 NPI symptoms.  **ADL ability** maintained in intervention group.  Mean change (SD):  ADL: Intervention 0.1 (0.6), Control -0.8 (1.0)  IADL: Intervention 0.5 (1.7), 0.9 (2.3) | Reported in related paper Moretti et al., 2002 ^60^:  **Caregiver stress** (RSS mean change)  Intervention -8.5 (5.4), p<.05 compared to baseline.  Control 3.3 (3.9), p <.01 group comparison.  Significant reduction in caregiver stress in intervention group. |
| *2^nd^ generation antipsychotics*  Rocca, 2007  ^61^ | To compare the efficacy and safety of 3 second generation antipsychotics, risperidone, olanzapine and quetiapine, when prescribed to treat behavioural/neuropsychiatric symptoms in dementia. Awareness assessed as a secondary aim. | Measure: CIRS (4 awareness subdomains). Used the original scoring for this measure, higher scores indicate greater impairment.  **Awareness Condition**  Risperidone Pre: 1.27 (0.46), post: 1.55 (0.51)  Olanzapine Pre: 1.25 (0.68), post: 1.38 (0.50)  Quetiapine Pre: 1.30 (0.66), post: 1.30 (0.47)  ANOVA n/s effect group or time, no interaction.  **Awareness Cognition**  Risperidone Pre: 1.27 (0.46), post: 1.55 (0.51)  Olanzapine Pre: 1.13 (0.62), post: 1.62 (0.50)  Quetiapine Pre: 1.40 (0.68), post: 1.60 (0.50)  ANOVA n/s effect group or interaction.  Time, F (5,115) = 10.009, p = 0.002,  awareness worsening.  **Awareness Disease progression**  Risperidone Pre: 1.55 (0.51), post:1.82 (0.39)  Olanzapine Pre: 1.62 (0.72), post: 1.75 (0.45)  Quetiapine Pre: 1.60 (0.50), post: 1.80 (0.41)  ANOVA: n/s effect group or interaction.  Time, F (5,115) = 4.528, p = 0.036, awareness worsening.  **Awareness Functional ability**  ANOVA: n/s effect group or time, no interaction  Risperidone Pre: 1.27 (0.63), post: 1.36 (0.66)  Olanzapine Pre: 1.25 (0.68), post 1.43 (0.51)  Quetiapine Pre: 1.50 (0.51), post: 1.50 (0.69) | **Neuropsychiatric symptoms (**NPI)  Total and item scores n/s group effect or interaction.  Significant difference over time all groups for total score and very significant for 3 NPI symptoms.  NPI total score:  ANOVA time, F (5,115) =81.837, p=0.000.  Delusions:  ANOVA time, F (5,115) = 34.163, p = 0.000, Hallucinations:  ANOVA time, F (5,115) = 26.422, p = 0.000, Agitation/aggression:  ANOVA time, F (5,115) = 16.202, p = 0.000.  Meaningful change in NPI total mean scores  Risperidone baseline 28.00 (5.44) reduced to 15.82 (4.68)  Olanzapine baseline 25.50 (6.61) reduced to 13.88 (4.66).  Quetiapine baseline 25.20 (7.85) reduced to 16.80 (7.88).  **Cognition** (MMSE)  N/s main effects of group or time, or interaction.  **ADL ability**  Significant improvements in all groups.  ADL: ANOVA time, F (5,115) = 11.171, p=.001  IADL: ANOVA time, F (5,115) =5.279, p=.023 | Not measured |

**Effect sizes** used in the studies: **ηp2** ^54,55,58^ negligible effect <0.01, small effect 0.01 to 0.05, medium effect 0.06 to 0.13, large effect ≥0.14. **Cohen’s d** ^56^ negligible effect <0.2, small effect 0.2 to 0.49, medium effect 0.5 to 0.79, large effect > 0.8.

**Abbreviations**: ADAS-Cog Scale Alzheimer's Disease Assessment Cognitive subscale. ADCS-ADL Alzheimer’s Disease Cooperative Study - Activities of Daily Living. ADL Activities of Daily Living index. ASPIDD Assessment Scale of Psychosocial Impact of the Diagnosis of Dementia. BASOLL Behavioural Assessment Scale of Later Life. BASQID Bath Assessment of Subjective Quality of Life. BEHAVE-AD Behavioral Pathology in Alzheimer's Disease. CIRS Clinical Insight Rating Scale. CSSD Cornell scale for Depression in Dementia. EQ-5D EuroQol-5 Domain Quality of Life. FAB Frontal Assessment Battery. GADS Guy’s Advanced Dementia Schedule. GDS Geriatric Depression Scale. IADL Instrumental Activities of Daily Living Scale. MARS (MFS) Memory Awareness Rating Scale (Memory Function Scale). MMSE Mini-Mental State Examination. MT86-1α Montreal-Toulouse Protocol 86 module 1α. NPI Neuropsychiatric Inventory. NPI-NH Neuropsychiatric Inventory nursing home. n/s non-significant. PRS Positive Response Schedule. QoL-AD Quality of Life in Alzheimer’s Disease scale. QUALID Quality of Life in Late-stage Dementia scale. SCQ Self-Consciousness Questionnaire. TPC Ten-Point Clock drawing test.

**Supplementary Table 4.** Awareness measures used in included studies.

| Study | Awareness measure | Description | Comments |
| --- | --- | --- | --- |
| Arroyo-Anllo, 2013 ^53^ | Self-Consciousness Questionnaire  ^62^ | Clinician interview using a 14-item scale rated by a clinician, with additional information from an informant. It has a broad view of awareness, with a subscale (‘anosognosia’) which more specifically assesses awareness of condition, awareness of memory problems, and awareness of difficulties. This subscale is more consistent with the concept of awareness we are using. Higher scores indicate better awareness | Presented raw scores for the anosognosia subscale. |
| Bertrand, 2023 ^54^ | Assessment Scale of Psychosocial Impact of the Diagnosis of Dementia  ^63^ | A 30-item discrepancy questionnaire covering four domains i.e. awareness of activities of daily living, cognitive functioning and health condition, emotional state, and social functioning and relationships, using the discrepancy between participant self-ratings and informant ratings as an indication of awareness. Higher scores indicate more impaired awareness. |  |
| Clare, 2013 ^56^ | AwareCare  ^64^ | Observational tool for care home staff to observe residents in a public area, during period of wakefulness for intervals of 10 minutes minimum. Observer records number and type of stimuli, spontaneous or introduced, and the number and type of responses observed, to create an individual responsiveness index, reflecting sensory awareness. | This study trained staff to use the tool, to investigate other outcomes of the staff training intervention. |
| Gueib, 2020 ^57^ | Self-Consciousness Questionnaire  ^62^ | Self-Consciousness Questionnaire as above. | Presented corrected scores for the anosognosia subscale. |
| Hilgeman, 2010 ^58^ | Memory Awareness Rating Scale- Memory Function Scale ^65^ | A 13-item discrepancy questionnaire, with everyday memory functioning rated by the participant and the informant. Higher ratings indicate better perceived ability; a higher discrepancy indicates lower awareness. |  |
| Moretti, 2002 ^59^ | Clinical Insight Rating Scale ^66^ | A 4-item clinician rating scale, using information from the person with dementia and an informant. Awareness rated regarding reason for clinic visit, of cognitive problems, of functional difficulties, and of progression of disease. | This study reverse-scored the scale, with lower scores indicating worse symptoms. The original scale has higher scores for more impaired awareness. |
| Rocca, 2007 ^61^ | Clinical Insight Rating Scale ^66^ | Clinical Insight Rating Scale as above. | Used the original scale scoring with higher scores for more impaired awareness |

**Supplementary Table 5.** TIDieR Checklist to rate quality of reporting of interventions.

| **Author (year)** | **1.**  **Brief name** | **2.**  **Why** | **3.**  **What materials** | **4.**  **What procedure** | **5.**  **Who provided** | **6.**  **How** | **7.**  **Where** | **8.**  **When and how much** | **9. Tailoring** | **10. Modification** | **11.**  **How well (planned)** | **12.**  **How well (actual)** | **TOTAL (max score 12)** | **% score** |
| --- | --- | --- | --- | --- | --- | --- | --- | --- | --- | --- | --- | --- | --- | --- |
| Arroyo-Anllo, 2013 ^53^ | 1 | 1 | 1 | 0.5 | 1 | 1 | 1 | 1 | 0 | 0 | 0 | 1 | 8.5 | 70.8 |
| Bertrand, 2023 ^54^ | 1 | 1 | 1 | 1 | 1 | 1 | 0.5 | 1 | 1 | 0 | 0 | 0 | 8.5 | 70.8 |
| Clare, 2013 ^56^ | 1 | 1 | 1 | 1 | 0.5 | 1 | 1 | 1 | 0 | 0 | 0 | 1 | 8.5 | 70.8 |
| Gueib, 2020 ^57^ | 1 | 1 | 1 | 1 | 1 | 1 | 1 | 1 | 0.5 | 0 | 0 | 0 | 8.5 | 70.8 |
| Hilgeman, 2010 ^58^ | 1 | 1 | 1 | 1 | 1 | 1 | 1 | 1 | 0 | 1 | 1 | 1 | 11 | 91.7 |
| Moretti, 2002 ^59^ | 1 | 1 | 1 | 1 | 1 | 1 | 0 | 1 | 0 | 0 | 0 | 0 | 7 | 58.3 |
| Rocca, 2007 ^61^ | 1 | 1 | 1 | 0.5 | 1 | 1 | 1 | 0.5 | N/A | N/A | N/A | N/A | 7 (out of 8) | 87.5 |

Ratings: 1 adequately described; 0.5 partial information; 0 Not reported or insufficient information for replication; N/A Not applicable.

**REFERENCES**

1. Brown E, Kay M, McDonald A. Post diagnostic education and support bolted on to an existing cognitive stimulation therapy programme. *Psychology of Older People: The FPOP Bulletin*. 2017;1(137):59-63. doi:10.53841/bpsfpop.2017.1.137.59

2. Clare L. *Intervening with everyday memory problems in early Alzheimer's*. Psychologists' Special Interest Group in Elderly People - PSIGE; 1999.

3. Franklin M, Lagnado D, Min CH, Mathur A, Kawsar F. Designing memory aids for dementia patients using earables. In: *Adjunct Proceedings of the 2021 ACM International Joint Conference on Pervasive and Ubiquitous Computing and Proceedings of the 2021 ACM International Symposium on Wearable Computers*. Assoc Computing Machinery; 2021:152-157.

4. Jones G, & Miesen, B. (Eds.). *Care-giving in dementia: research and applications: volume 2*. Routledge; 1997. doi:10.4324/9781315800141

5. Miesen BM, & Jones, G. M. (Eds.). *Care-giving in dementia: research and applications, vol. 4*. Routledge; 2006. doi:10.1017/s0144686x0700640x

6. Buchwitz TM, Maier F, Greuel A, Eggers C. Improving self-awareness of motor symptoms in patients with Parkinson's disease by using mindfulness - a study protocol for a randomized controlled trial. *Front Psychol*. 2020;11:13. doi:10.3389/fpsyg.2020.00743

7. Arber D. *Aids to awareness and communication*. Vol. 3. 1987. *Computer Applications in Social Work and Allied Professions*. 0267 1980.

8. Gotell E, Brown S, Ekman S-L. Influence of caregiver singing and background music on posture, movement, and sensory awareness in dementia care. *Int Psychogeriatr*. 2003;15(4):411-430. doi:10.1017/S1041610203009657

9. Tabak N, Bergman R, Alpert R. The mirror as a therapeutic tool for patients with dementia. *Int J Nurs Pract*. 1996;2(3):155-159. doi:10.1111/j.1440-172x.1996.tb00042.x

10. Vespa A, Fabbietti P, Giulietti MV. Study of the effects of mindfulness training on quality of life of patients with Alzheimer's disease and their caregivers (Dyad Mindfulness Project). *Aging Clin Exp Res*. 2022;34(1):65-71. doi:10.1007/s40520-021-01907-x

11. Barnes D, Lee A, Lee J, et al. Moving together livestream virtual group movement classes for persons with dementia and caregivers: feasibility and satisfaction. *J Am Geriatr Soc*. 2020;68:S202. doi:10.1111/jgs.16431

12. Barnes D, Lee J, Nicosia F, et al. Preventing loss of independence through exercise (PLIE) for persons with dementia: a randomized, controlled trial. *J Am Geriatr Soc*. 2020;68:S139. doi:10.1111/jgs.16431

13. Department of Health. *Transforming the quality of dementia care. Consultation on a National Dementia Strategy*. Department of Health; 2008. <http://www.cpa.org.uk/cpa/consultation_on_national_dementia_strategy.pdf>

14. Department of Health. *Living well with dementia: a national dementia strategy*. 2009. doi:10.1037/e608082011-001

15. Dombrowski W, Mims A, Kremer I, et al. Dementia ideal care: ecosystem map of best practices and care pathways enhanced by technology and community. *J Alzheimers Dis*. 2024;100(1):87-117. doi:10.3233/JAD-231491

16. Francis-Coad J, Weselman T, Burton E, Beilby J, Hill AM. Fall prevention education co-designed and evaluated with community-dwelling older people living with dementia and their caregivers: a feasibility study. *Health Soc Care Community*. 2023;2023:13. doi:10.1155/2023/9965507

17. NICE National Institute for Health and Care Excellence. *Dementia: QS 184*. National Institute for Health and Care Excellence; 2019. <https://www.nice.org.uk/guidance/qs184>

18. Pin Tan H, Hsiao KY, Ouyang WC. Traditional Chinese medicine improves circadian rhythm disturbance of an elder with dementia: a case report. *Int Psychogeriatr*. 2019;31:118. doi:10.1017/S1041610219001339

19. Xavier FM, Abidin WZWN, Hamzah F, Hashim A, Nordin N. Cognitive stimulation therapy in mild to moderate dementia in Hospital Sungai Buloh. *Med J Malaysia*. 2022;77(Supplement 3), <https://www.e-mjm.org/2022/v77s3/OR-05-06.pdf>

20. Bahro M. Giving up driving in Alzheimer's Disease - an integrative therapeutic approach. *Int J Geriatr Psychiatry*. 1995;10(10):871-874. doi:10.1002/gps.930101010

21. Berenbaum R, Tziraki C, Baum R, et al. Focusing on emotional and social intelligence stimulation of people with dementia by playing a serious game-proof of concept study. *Front Comput Sci-Switz*. 2020;2:15. doi:10.3389/fcomp.2020.536880

22. Burns A, Guthrie E, Marino-Francis F. Brief psychotherapy in Alzheimer's disease. *Br J Psychiatry*. 2005;187:143-7. doi:10.1192/bjp.187.2.143

23. Hertel PA, Schier Anzelmo NA, Tomaszewski-Farias SE. Equine guided support: workshops for people living with early stage dementia and their care partners. *Alzheimers Dement*. 2019;15:P1172. doi:10.1016/j.jalz.2019.06.3561

24. Lantz MS, Buchalter EN, McBee L. The Wellness group: a novel intervention for coping with disruptive behavior in elderly nursing home residents. *Gerontologist*. 1997;37(4):551-6. doi:10.1093/geront/37.4.551

25. Marco P, Redolat R. Alzheimer's disease, grieving process, and art therapy: case study. *Art Ther*. 2023;40(1):40-45. doi:10.1080/07421656.2022.2066951

26. Morgan DG. Evaluating rural nursing home environments: dementia special care units versus integrated facilities. *Aging Ment Health*. 2004;8(3):256-265. doi:10.1080/1360786041000166796

27. Nicosia FM, Lee JA, Chesney MA, et al. Adaptation of an in-person mind-body movement program for people with cognitive impairment or dementia and care partners for online delivery: feasibility, satisfaction and participant-reported outcomes. *Glob Adv Integr Med Health*. 2023;12:27536130231202989. doi:10.1177/27536130231202989

28. Oksnebjerg L, Woods B, Vilsen CR, et al. Self-management and cognitive rehabilitation in early stage dementia - merging methods to promote coping and adoption of assistive technology. A pilot study. *Aging Ment Health*. 2019:1-10. doi:10.1080/13607863.2019.1625302

29. Punitha P, Shrishte AS. The efficacy of prospective memory and metacognitive skills training in improving the cognitive skills and the quality of life of elderly persons with dementia. *Cureus J Med Sci*. 2024;16(8):8. doi:10.7759/cureus.67454

30. Sedigh M, Mosalanejad L, Bazrafkan L, Mohsenzadeh M. Memory rehabilitation in aging as a need for today's modern societies: designing and determining the effects of memory-boosting mobile application on the cognitive function of aging with cognitive dysfunction. *Ageing Int*. 2024;49(2):417-433. doi:10.1007/s12126-023-09551-8

31. Steiner GZ, George ES, Metri NJ, et al. Use of complementary medicines and lifestyle approaches by people living with dementia: exploring experiences, motivations and attitudes. *Int J Older People Nurs*. 2021;16(5):10. doi:10.1111/opn.12378

32. Bonney KR, Almeida OP, Flicker L, et al. Reduced awareness of executive dysfunction in Alzheimer's disease is associated with increased carer burden. *Int Psychogeriatr*. 2007;19(6):1173-1175. doi:10.1017/S1041610207005947

33. Cotrell V, Wild K, Bader T. Medication management and adherence among cognitively impaired older adults. *J Gerontol Soc Work*. 2006;47(3-4):31-46. doi:10.1300/J083v47n03_03

34. Niehoff D, Dodge S, Graham E, et al. Insights on FTD: an externally-led patient-focused drug development meeting (P8-3.004). *Neurology*. 2022;98(18_supplement):663. doi:10.1212/WNL.98.18_supplement.663

35. Ross SD, Kress J, Rodriguez FS. Self-perceived problems in daily activities and strategy building in people with different stages of dementia. *Psychogeriatrics*. 2024;24:108-116. doi:10.1111/psyg.13047

36. Silva AR, Pinho MS, Macedo L, Souchay C, Moulin C. Mnemonic anosognosia in Alzheimer's disease is caused by a failure to transfer online evaluations of performance: evidence from memory training programs. *J Clin Exp Neuropsychol*. 2017;39(5):419-433. doi:10.1080/13803395.2016.1231799

37. Au A. Connecting through caregiving: perspective-taking interventions for adult child caregivers of persons living with dementia. *Age Ageing*. 2023;52:i3. doi:10.1093/ageing/afac322.011

38. Berk L, Warmenhoven F, Stiekema APM, et al. Mindfulness-based intervention for people with dementia and their partners: results of a mixed-methods study. *Front Aging Neurosci*. 2019;11:10. doi:10.3389/fnagi.2019.00092

39. Binetti G. Predictors of comprehensive stimulation program efficacy in patients with cognitive impairment. Clinical practice recommendations. *Int J Geriatr Psychiatry*. 2013;28(1):26-33. doi:10.1002/gps.3785

40. Clare L, Wilson BA, Carter G, Roth I, Hodges JR. Relearning face-name associations in early Alzheimer's disease. *Neuropsychology*. 2002;16(4):538-547. doi:10.1037//0894-4105.16.4.538

41. Clare L, Wilson BA, Carter G, Roth I, Hodges JR. Awareness in early-stage Alzheimer's disease: relationship to outcome of cognitive rehabilitation. *J Clin Exp Neuropsychol*. 2004;26(2):215-226. doi:10.1076/jcen.26.2.215.28088

42. Contador I, Fernandez-Calvo B, Ramos F, Mograbi DC, Morris RG. Interaction effect of awareness and educational attainment on the benefits of multicomponent intervention for persons with mild Alzheimer's disease. *Arch Clin Neuropsychol*. 2016;31(8):1037-1042. doi:10.1093/arclin/acw074

43. de Farias JM, Tramontin ND, Pereira EV, et al. Physical exercise training improves judgment and problem-solving and modulates serum biomarkers in patients with Alzheimer's disease. *Mol Neurobiol*. 2021:9. doi:10.1007/s12035-021-02411-z

44. Dutzi I, Schwenk M, Kirchner M, Bauer JM, Hauer K. "What would you like to achieve?" Goal-setting in patients with dementia in geriatric rehabilitation. *BMC Geriatr*. 2019;19:280. doi:10.1186/s12877-019-1296-7

45. Geurten M, Salmon E, Willems S, Bastin C. Boosting familiarity-based memory decisions in Alzheimer's disease: the importance of metacognition. *J Int Neuropsychol Soc*. 2021;27(3):239-248. doi:10.1017/S1355617720000910

46. Kelly M, Finan S, Lawless M, et al. An evaluation of community-based cognitive stimulation therapy: a pilot study with an Irish population of people with dementia. *Ir J Psychol Med*. 2017;34(3):157-167. doi:10.1017/ipm.2016.23

47. Koltai DC, Welsh-Bohmer KA, Schmechel DE. Influence of anosognosia on treatment outcome among dementia patients. *Neuropsychol Rehabil*. 2001;11(3):455-475. doi:10.1080/09602010042000097

48. Martinez-Moreno M, Cerulla N, Chico G, Quintana M, Garolera M. Comparison of neuropsychological and functional outcomes in Alzheimer's disease patients with good or bad response to a cognitive stimulation treatment: a retrospective analysis. *Int Psychogeriatr*. 2016;28(11):1821-1833. doi:10.1017/S104161021600123X

49. Salmon E, Lekeu F, Quittre A, et al. Awareness and cognitive rehabilitation in Alzheimer's disease and frontotemporal dementia. *Alzheimers Dement*. 2024;10(2)e12469. doi:10.1002/trc2.12469

50. Schapira M, Gonzalez Salvia M, Perman G, et al. Integrated care for patients with dementia and behavioral disorders in the home setting. *J Am Geriatr Soc*. 2023;71:S171. doi:10.1111/jgs.18336

51. Villars H, Dupuy C, Soler P. A follow-up intervention in severely demented patients after discharge from a special Alzheimer acute care unit. *Int J Geriatr Psychiatry*. 2013;28(11):1131-40. doi:10.1002/gps.3932

52. Yokoi T, Okamura H, Yamamoto T, et al. Effect of wearing fingers rings on the behavioral and psychological symptoms of dementia: an exploratory study. *SAGE Open Med*. 2017;5 2050312117726196. doi:10.1177/2050312117726196

53. Arroyo-Anllo EM, Diaz JP, Gil R. Familiar music as an enhancer of self-consciousness in patients with Alzheimer's disease. *Biomed Res Int*. 2013;2013:752965. 752965. doi:10.1155/2013/752965

54. Bertrand E, Marinho V, Naylor R, et al. Metacognitive improvements following cognitive stimulation therapy for people with dementia: evidence from a pilot randomized controlled trial. *Clin Gerontol*. 2023;46(2):267-276. doi:10.1080/07317115.2022.2155283

55. Marinho V, Bertrand E, Naylor R, et al. Cognitive stimulation therapy for people with dementia in Brazil (CST-Brasil): results from a single blind randomized controlled trial. *Int J Geriatr Psychiatry*. 2021;36(2):286-293. doi:10.1002/gps.5421

56. Clare L, Whitaker R, Woods RT, et al. AwareCare: a pilot randomized controlled trial of an awareness-based staff training intervention to improve quality of life for residents with severe dementia in long-term care settings. *Int Psychogeriatr*. 2013;25(1):128-139. doi:10.1017/S1041610212001226

57. Gueib C, Pop A, Bannay A, et al. Impact of a healing garden on self-consciousness in patients with advanced Alzheimer's disease: an exploratory study. *J Alzheimers Dis*. 2020;75:1283-1300. doi:10.3233/JAD-190748

58. Hilgeman MM. *Preserving identity and planning for advance care (PIPAC): an intervention to increase coping in the early stages of dementia*. Dissertation/Thesis. 2010.

59. Moretti R, Torre P, Antonello RM, Cazzato G, Griggio S, Bava A. An open-label pilot study comparing rivastigmine and low-dose aspirin for the treatment of symptoms specific to patients with subcortical vascular dementia. *Curr Ther Res Clin Exp*. 2002;63(7):443-458. doi:10.1016/S0011-393X(02)80050-9

60. Moretti R, Torre P, Antonello RM, Cazzato G, Bava A. Rivastigmine in subcortical vascular dementia: an open 22-month study. *J Neurol Sci*. 2002;203-204:141-146. doi:10.1016/S0022-510X(02)00280-0

61. Rocca P, Marino F, Montemagni C, Perrone D, Bogetto F. Risperidone, olanzapine and quetiapine in the treatment of behavioral and psychological symptoms in patients with Alzheimer's disease: preliminary findings from a naturalistic, retrospective study. *Psychiatry Clin Neurosci*. 2007;61(6):622-629. doi:10.1111/j.1440-1819.2007.01729.x

62. Gil R, Arroyo-Anllo E, Ingrand P, et al. Self-consciousness and Alzheimer's disease. *Acta Neurol Scand*. 2001;104(5):296-300. doi:10.1034/j.1600-0404.2001.00280.x

63. Dourado MCN, Mograbi DC, Santos RL, et al. Awareness of disease in dementia: factor structure of the Assessment Scale of Psychosocial Impact of the Diagnosis of Dementia. *J Alzheimers Dis*. 2014;41(3):947-956. doi:10.3233/jad-140183

64. Clare L, Whitaker R, Quinn C, et al. AwareCare: development and validation of an observational measure of awareness in people with severe dementia. *Neuropsychol Rehabil*. 2012;22(1):113-133. doi:10.1080/09602011.2011.640467

65. Clare L, Wilson BA, Carter G, Roth I, Hodges JR. Assessing awareness in early-stage Alzheimer's disease: development and piloting of the Memory Awareness Rating Scale. *Neuropsychol Rehabil*. 2002;12(4):341-362. doi:10.1080/09602010244000129

66. Ott BR, Fogel BS. Measurement of depression in dementia: self vs clinician rating. *Int J Geriatr Psychiatry*. 1992;7(12):899-904. doi:10.1002/gps.930071209
